# 160. RSV Pre-F IgG levels in pediatric hematopoietic stem cell transplant (HCT) and leukemia/lymphoma patients

**DOI:** 10.1093/ofid/ofae631.046

**Published:** 2025-01-29

**Authors:** Alastair Murray, Erica Clark, Helen Y Chu, Janet A Englund

**Affiliations:** University of Washington/Seattle Children's, Seattle, WA; University of Washington, Seattle, Washington; University of Washington, Seattle, Washington; Seattle Children’s Hospital, Seattle, Washington

## Abstract

**Background:**

Hematopoietic stem cell transplant (HCT) patients are at increased risk of morbidity from RSV infection. Vaccines and monoclonal antibodies directed against the RSV pre-fusion surface protein are now licensed for protection of infants against RSV. Analysis of RSV-specific antibody seroprevalence in pediatric HCT and oncology patients will help identify those who may benefit from receipt of an RSV monoclonal antibody, such as nirsevimab, for protection against severe disease.

**Table 1.**
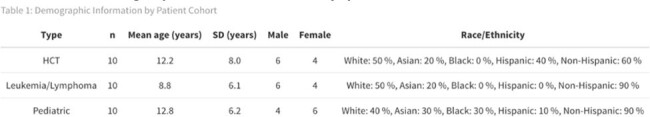
Demographic information by patient cohort

**Methods:**

This prospective study collected deidentified serum samples from pediatric (age 0-24 years) HCT patients (n = 10), pediatric leukemia and lymphoma (LL) patients (without blood transfusion < 60 days) (n = 10) and general pediatric patients without immunocompromising conditions or respiratory illness evaluated in the emergency room (n = 10) in Seattle, WA, USA. Samples were collected at time of transplant between February-April 2024 and concurrently collected from the two other cohorts. Samples were tested for quantitative RSV pre-fusion IgG concentration using an ELISA assay. One LL patient received nirsevimab (excluded from analysis). Values were natural log-transformed before all statistical processes and comparisons were performed by ANOVA testing.

**Figure 1.**
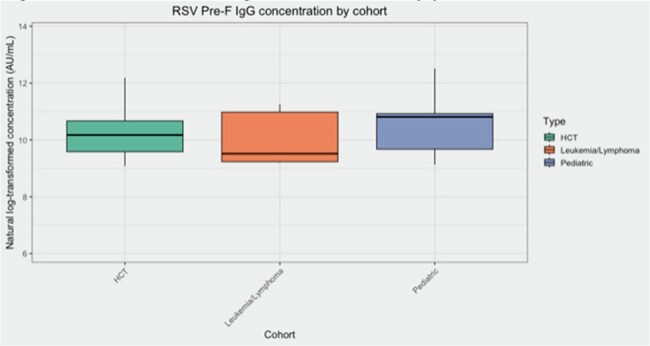
RSV Pre-fusion IgG concentration by patient cohort

**Results:**

Mean age was similar among HCT and general pediatrics cohorts (12.2 years versus 12.8) and slightly younger among the LL cohort (8.8) (ANOVA, p = 0.38) (Table 1). Mean log antibody concentration was similar across all three cohorts (BMT: 10.3 AU/mL, LL: 9.9, general pediatric: 10.5), (ANOVA, p = 0.62) (Figure 1). Mean log antibody concentration was also similar by age group (0-5 years: 10.1 AU/mL, 6-12 years: 10.2, 13-24 years: 10.4), (ANOVA, p = 0.33) (Figure 2).

**Figure 2.**
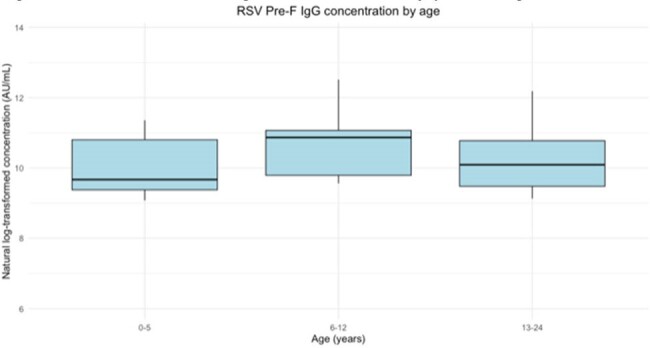
RSV Pre-fusion IgG concentration by patient age

**Conclusion:**

RSV pre-fusion IgG concentration was comparable across cohorts of pediatric HCT patients, leukemia/lymphoma and general pediatric patients. Further study enrollment is ongoing to capture changes in antibody level over the course of a respiratory viral season, along with assessment of RSV neutralizing antibody, which may provide a more robust correlate of protection. This information may inform the need for further clinical trial prophylaxis studies, given the vulnerability to RSV disease in these patient populations.

**Disclosures:**

**Helen Y. Chu, MD, MPH**, Abbvie: Advisor/Consultant|Merck: Advisor/Consultant|Vir: Advisor/Consultant **Janet A. Englund, MD**, Abbvie: Advisor/Consultant|AstraZeneca: Advisor/Consultant|AstraZeneca: Grant/Research Support|GlaxoSmithKline: Advisor/Consultant|GlaxoSmithKline: Grant/Research Support|Meissa Vaccines: Advisor/Consultant|Merck: Advisor/Consultant|Pfizer: Board Member|Pfizer: Grant/Research Support|Pfizer: Speaker at meeting|SanofiPasteur: Advisor/Consultant|Shinogi: Advisor/Consultant

